# Supplementary material for: Chromothripsis during telomere crisis is independent of NHEJ, and consistent with a replicative origin
Source: Genome Res. 2019 May;29(5):737–49. doi: 10.1101/gr.240705.118 (PMC6499312; doi:10.1101/gr.240705.118)
Supplement: Supplemental Material [file supp_gr.240705.118_Supplemental_file_1.zip › contigs/annotated_contigs/DB79/contig.2.DB79_length_473_mean_cov_10.600422833.docx]

**DB79_length_473_mean_cov_10.600422833**

```
ATACATGCGCCATGCTGGTGTGCTGCACCTATTAACTCGTCATTTAGCATTAGGTATATCTCCTAATGCTATCCCTCCCCCCTCCCCCT
 >chrY:59028951-59029132 + E=3e-79
ACCCCACATTGCCCTTGTCTCCCAGGCTGGAGAACAGTGGTGCTATCTCGGCTCACTGCAACCTCCGCCTCCTGGGTTCAAGCAATTCT

CC|T|ATATATATATAAAACAAGAGCTTACAGAACAGGCAGCACCAAGTGGGAGCTGGGGGGCTTGTCTTGATGTTGCATTTGATAAAT
   >chrY:59030574-59030867 + E=8e-153
ATACACCGTTTTTACTCAGATTATATTTTTGCTTAGATTTGGGCTAAAGATCCCCAAAGCTGCCCCTTGGTATTGACACTCATTAAGAA

ATTAAGGAAGAGATAGTACTATTGAACTATTTTCCAAAAAAAGAAAAAAAAAAGACAAAAACCCAAAACAAATGGGATGTGATGTACAT

TTTCCATGTTATTTTATACATGAGCTATCT
```
